# Supplementary material for: Proteomic Analysis of Hydromethylthionine in the Line 66 Model of Frontotemporal Dementia Demonstrates Actions on Tau-Dependent and Tau-Independent Networks
Source: Cells. 2021 Aug 22;10(8):2162. doi: 10.3390/cells10082162 (PMC8391171; doi:10.3390/cells10082162)

Supplementary Figure S1

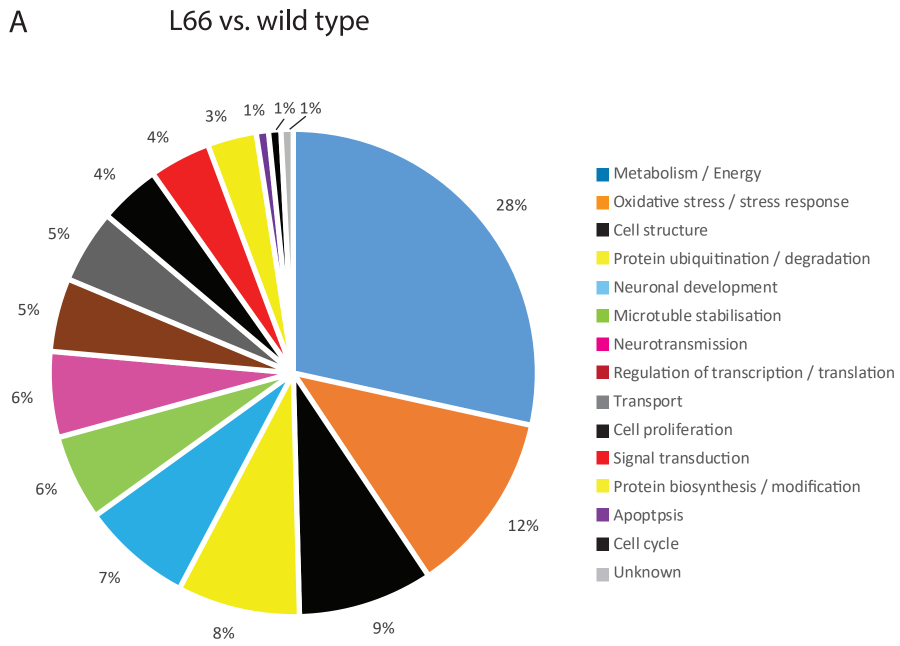

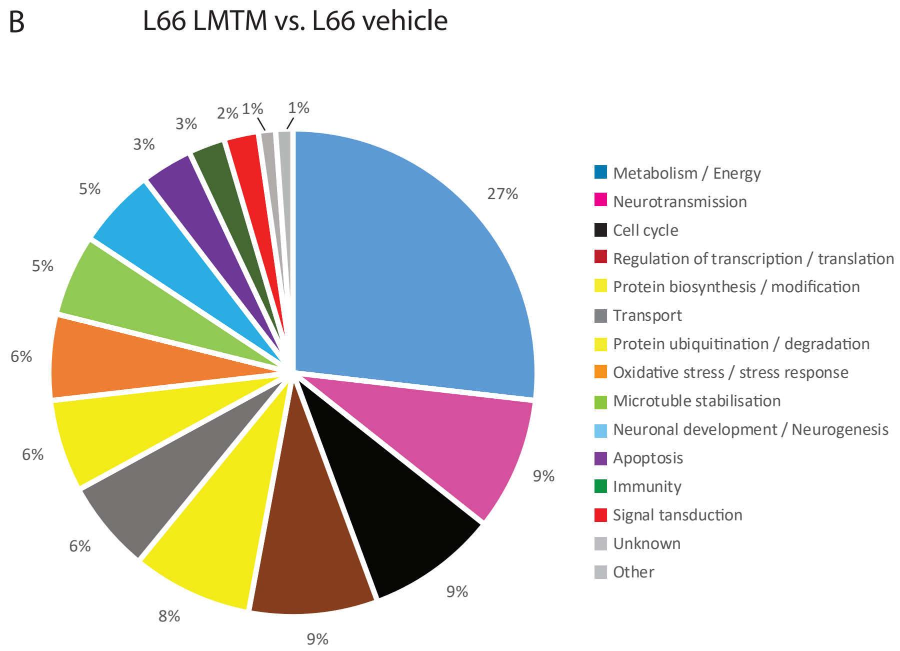

Supplementary Figure S2

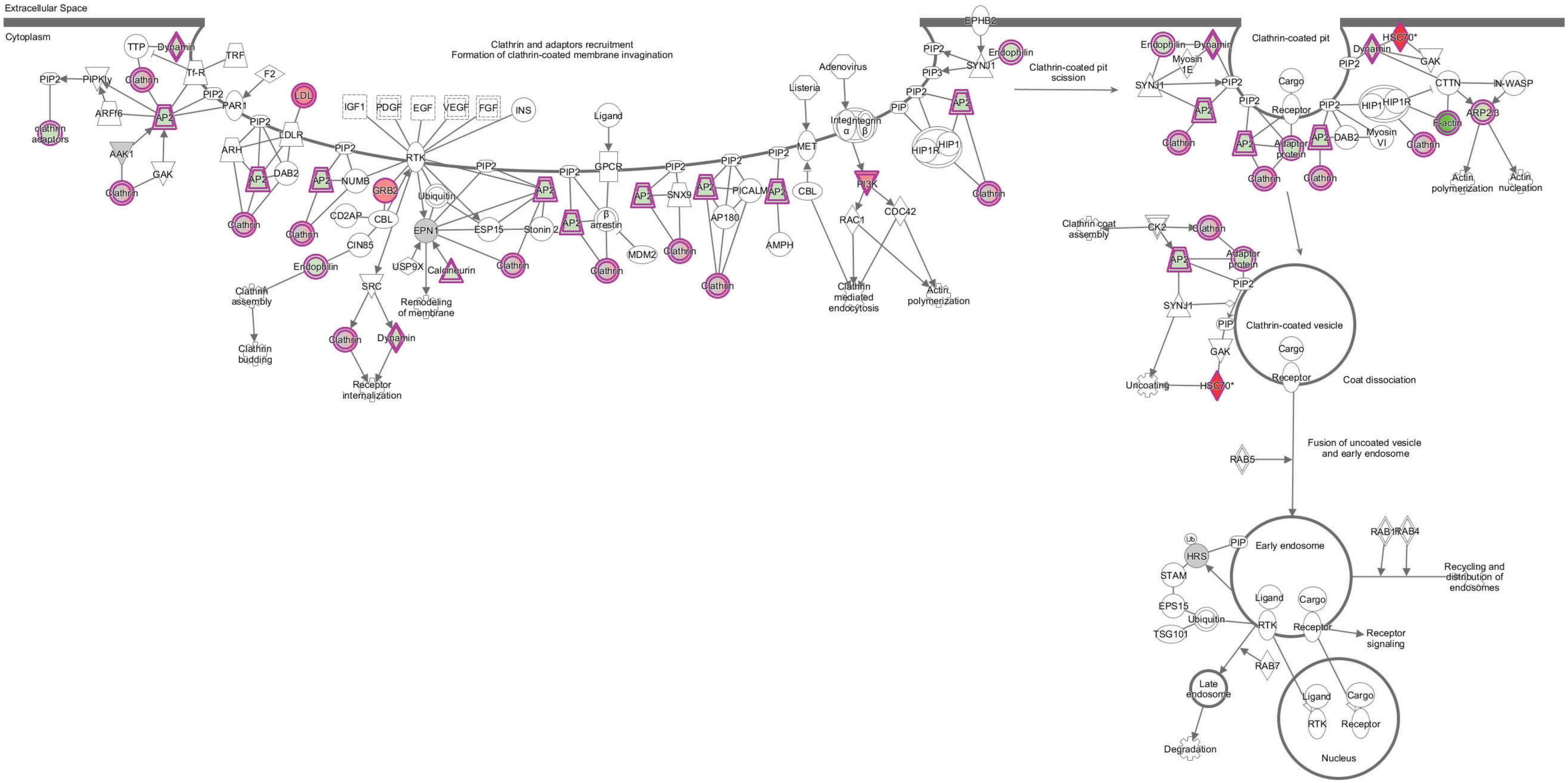

Supplementary Figure S3

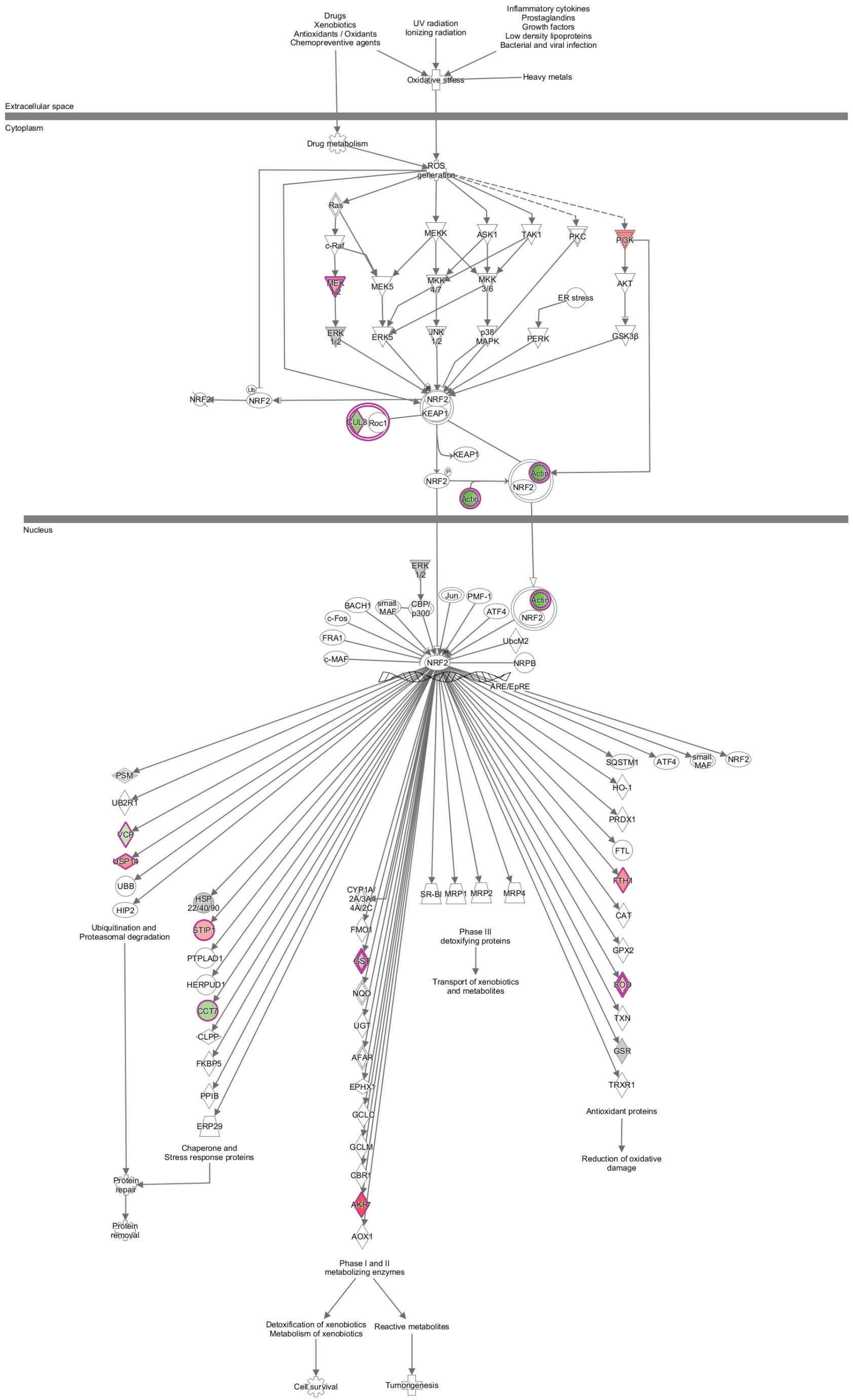

Supplementary Figure S4

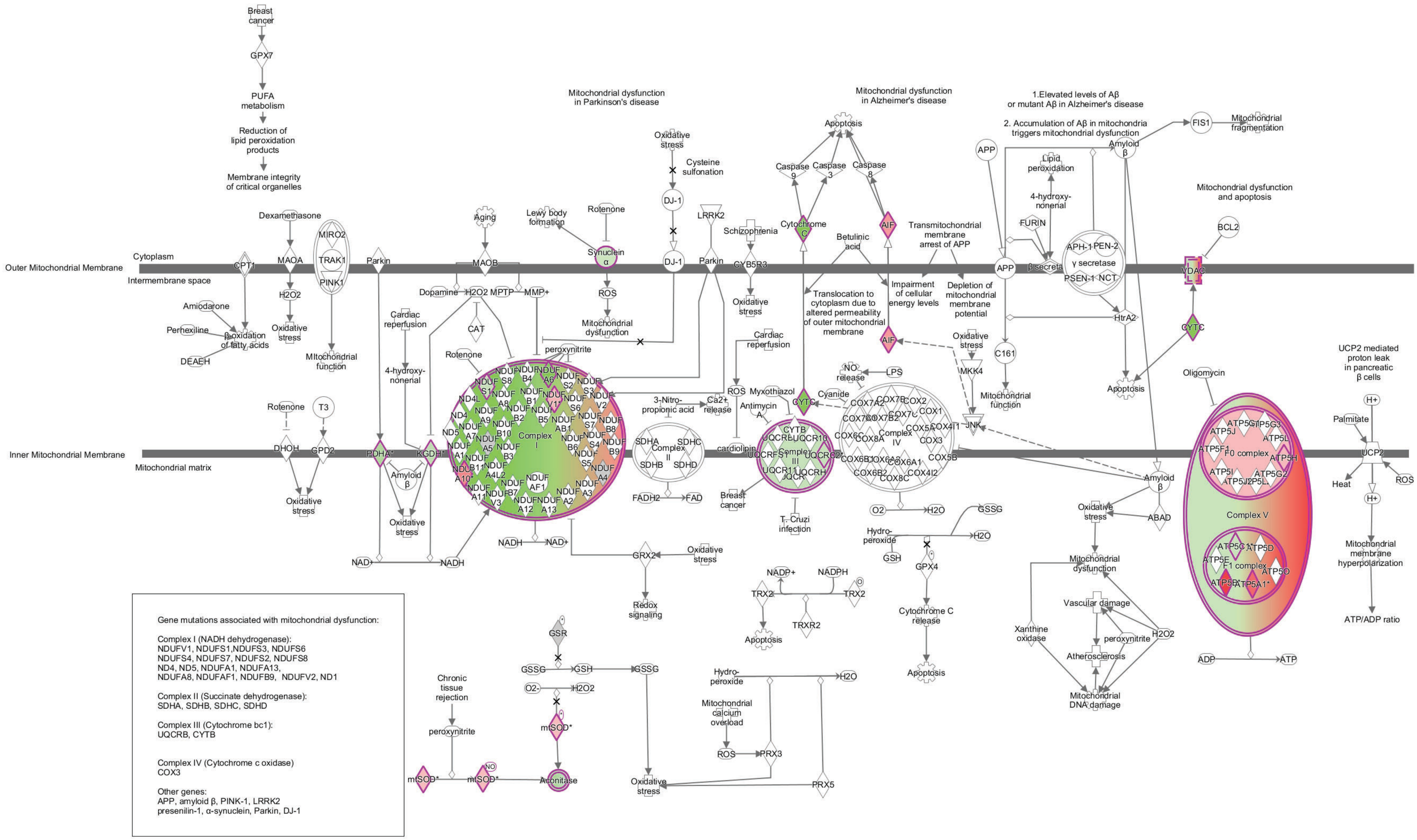

Supplementary Figure S5

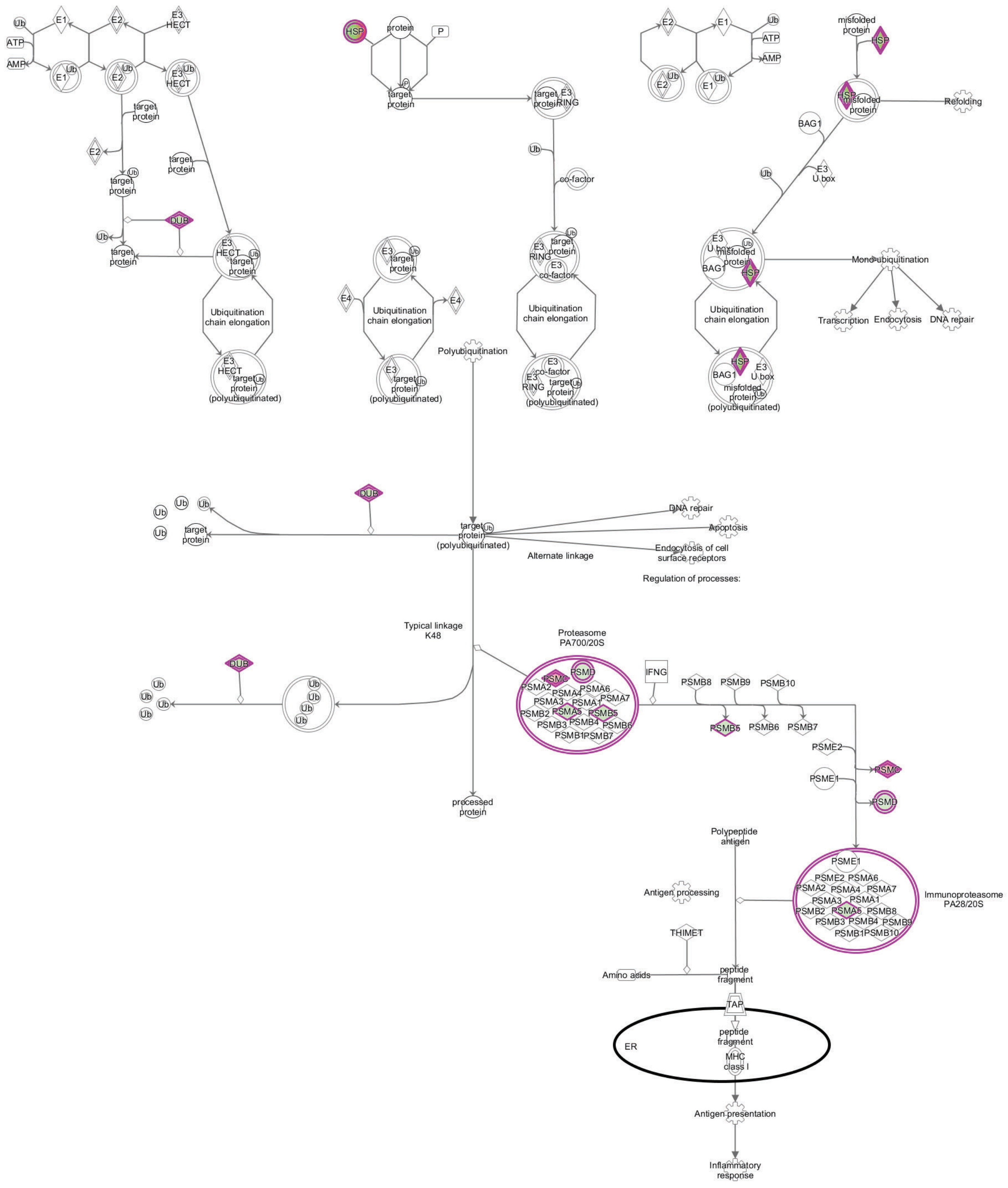

Supplement: Supplementary file 1 [file cells-10-02162-s001.zip › Schwab et al Supplementary Figures.pdf]
